# Effectiveness of Gastric Bypass Versus Gastric Sleeve for Cardiovascular Disease: Protocol and Baseline Results for a Comparative Effectiveness Study

**DOI:** 10.2196/14936

**Published:** 2020-04-06

**Authors:** Karen J Coleman, Heidi Fischer, David E Arterburn, Douglas Barthold, Lee J Barton, Anirban Basu, Anita Courcoulas, Cecelia L Crawford, Peter Fedorka, Benjamin Kim, Edward Mun, Sameer Murali, Kristi Reynolds, Kangho Suh, Rong Wei, Tae K Yoon, Robert Zane

**Affiliations:** 1 Department of Research and Evaluation Kaiser Permanente Southern California Pasadena, CA United States; 2 Health Research Institute Kaiser Permanente Washington Seattle, WA United States; 3 The Comparative Health Outcomes, Policy, and Economics Institute Department of Pharmacy University of Washington Seattle, WA United States; 4 Department of Surgery School of Medicine University of Pittsburgh Pittsburgh, PA United States; 5 Regional Nursing Research Program Kaiser Permanente Southern California Pasadena, CA United States; 6 Department of Surgery San Bernardino Medical Center Kaiser Permanente Southern California Ontario, CA United States; 7 Department of Surgery South Bay Medical Center Kaiser Permanente Southern California Harbor City, CA United States; 8 Center for Healthy Living San Bernardino Medical Center Kaiser Permanente Southern California Fontana, CA United States

**Keywords:** race, weight loss surgery, integrated health care system

## Abstract

**Background:**

When compared with conventional weight loss strategies, bariatric surgery results in substantially greater durable weight loss and rates of disease remission.

**Objective:**

The ENGAGE CVD (Effectiveness of Gastric Bypass versus Gastric Sleeve for Cardiovascular Disease) cohort study aimed to provide population-based, comprehensive, rigorous evidence for clinical and policy decision making regarding the choice between gastric bypass and gastric sleeve for overall cardiovascular disease (CVD) risk reduction, risk factor remission, and safety.

**Methods:**

The cohort had 22,095 weight loss surgery patients from a large integrated health care system in Southern California assembled from 2009 to 2016 who were followed up through 2018. Bariatric surgery patients were followed up for the length of their membership in the health care system. Of the patients who had at least five years of follow-up (surgery between 2009 and 2013), 85.86% (13,774/16,043) could contribute to the outcome analyses for the ENGAGE CVD cohort.

**Results:**

Patients in the ENGAGE CVD cohort were 44.6 (SD 11.4) years old, mostly women (17,718/22,095; 80.19%), with 18.94% (4185/22,095) non-Hispanic black and 41.80% (9235/22,095) Hispanic, and had an average BMI of 44.3 (SD 6.9) kg/m^2^ at the time of surgery. When compared with patients who did not contribute data to the 5-year outcome analysis for the ENGAGE CVD cohort (2269/16,043; 14.14%), patients who contributed data (13,774/16,043; 85.86%) were older (*P*=.002), more likely to be women (*P*=.02), more likely to be non-Hispanic white (*P*<.001), more likely to have had an emergency department visit in the year before surgery (*P*=.006), less likely to have a mental illness before surgery (*P*<.001), and more likely to have had a CVD event at any time before surgery (*P*<.001).

**Conclusions:**

This study had one of the largest populations of gastric sleeve patients (n=13,459). The 5-year follow-up for those patients who had surgery between 2009 and 2013 was excellent for a retrospective cohort study at 85.86% (13,774/16,043). Unlike almost any study in the literature, the majority of the ENGAGE CVD cohort was racial and ethnic minority, providing a rare opportunity to study the effects of bariatric surgery for different racial and ethnic groups, some of whom have the highest rates of severe obesity in the United States. Finally, it also used state-of-the-art statistical and econometric comparative effectiveness methods to mimic the effect of random assignment and control for sources of confounding inherent in large observational studies.

**International Registered Report Identifier (IRRID):**

RR1-10.2196/14936

## Introduction

### Overview of Surgical Treatment for Severe Obesity

The prevalence of severe obesity (BMI >35 kg/m^2^) has increased over the past several decades. Rates are as high as 36% for middle-aged black women compared with 16% for their white counterparts in the United States [1]. Even with intensive, multicomponent lifestyle interventions, only 50% of studies show 5% weight loss (considered clinically meaningful), and most of the participants gain back at least half of this lost weight over 18 to 30 months [2]. These poor outcomes have resulted in the development of surgical treatments, referred to as bariatric surgery, for severe obesity. When compared with conventional weight loss strategies, bariatric surgery results in seven times the amount of weight loss and 15.8 times the rate of diabetes remission [3], and these differences remain up to 5 years [4,5]. Given the poor results from traditional weight loss methods [2], and the designation of obesity as a disease [6], bariatric surgery may become a more common treatment of choice for adults with severe obesity.

Two surgical treatments constitute most bariatric operations in the United States: vertical sleeve gastrectomy (VSG) and Roux-en-Y gastric bypass (RYGB). VSG, in which stomach size is reduced, was initially performed as the first part of a multistage procedure in 2000. RYGB, in which gastric capacity is also limited but with an additional bypass of the first few feet of small intestine, was first performed in 1994 [7]. VSG has emerged as the fastest growing bariatric operation in the United States. Between 2008 and 2014, there was a dramatic increase in VSGs from 4% to 51% of all bariatric operations, whereas RYGB declined from 51% to 27% [8]. The reasons for this shift have not been systematically studied, but based upon our own work [9], it is likely because of patients’ and surgeons’ perceptions that although VSG and RYGB have similar weight loss and disease remission, VSG is easier to perform with fewer complications compared with RYGB.

### Evidence for Comparative Effectiveness of Surgical Treatments

Unfortunately, the use of VSG has outpaced a rigorous evidence base for its comparative effectiveness to RYGB [10-15]. In addition, few large population-based studies in real-world health care settings have adequate methodological rigor to account for the fact that VSG and RYGB operations are not randomly assigned. Patients with risk factors for cardiovascular disease (CVD), especially type 2 diabetes mellitus (T2DM), are more likely to undergo RYGB [16]. The reasons for this are not clear; however, it is likely that surgeons and patients believe RYGB is more effective than VSG for resolving T2DM. If this treatment choice preference is not accounted for in the analyses, then erroneous conclusions could be made about the effectiveness of one operation compared with another because the patients receiving each treatment are different in ways that also affect the outcome.

### Addressing Limitations in the Evidence Base

Rigorous statistical methods such as matching, propensity scores, and/or instrumental variables have only been applied to the study of the comparative effectiveness of VSG and RYGB in the remission and relapse of T2DM. To our knowledge, there have been no rigorous comparative effectiveness studies published for other risk factors for CVD, including hypertension and dyslipidemia. In addition, there are no published studies on the comparative effectiveness of VSG and RYGB for reducing overall CVD risk beyond the first year after surgery. The ENGAGE CVD (Effectiveness of Gastric Bypass versus Gastric Sleeve for Cardiovascular Disease) cohort study was funded by the National Heart, Lung, and Blood Institute to provide population-based, comprehensive, rigorous evidence for clinical and policy decision making regarding the choice between RYGB and VSG for overall CVD risk reduction, risk factor remission, and safety. The ENGAGE CVD study uses state-of-the-art statistical and econometric comparative effectiveness methods, including propensity scores and local instrumental variables (LIVs), to mimic the effect of random assignment and control for sources of both observed and unobserved confounding inherent in large observational studies.

### Study Objectives and Hypotheses

There were three aims for the ENGAGE CVD study. Aim 1 compared the effectiveness of VSG and RYGB in remission and relapse of CVD risk factors and reduction in overall CVD risk. For this aim we hypothesized that RYGB patients would experience a higher rate of T2DM, hypertension, and dyslipidemia remission and lower rate of relapse compared with VSG patients. RYGB patients would also have a greater reduction in overall CVD risk. Aim 2 compared VSG and RYGB surgical safety. We hypothesized that VSG patients would have better short- and long-term safety outcomes than RYGB patients. Aim 3 was designed to understand the treatment effect heterogeneity in remission and relapse of CVD risk factors, reduction in overall CVD risk, and safety outcomes for patients with different racial and ethnic backgrounds, genders, ages, and disease burdens at the time of surgery. Based upon our own work in this area, we expected an interaction of racial and ethnic minority, male sex, older age, and having a higher disease burden in attenuating the differences hypothesized between RYGB and VSG.

## Methods

### Settings and Participants

**Figure 1:**
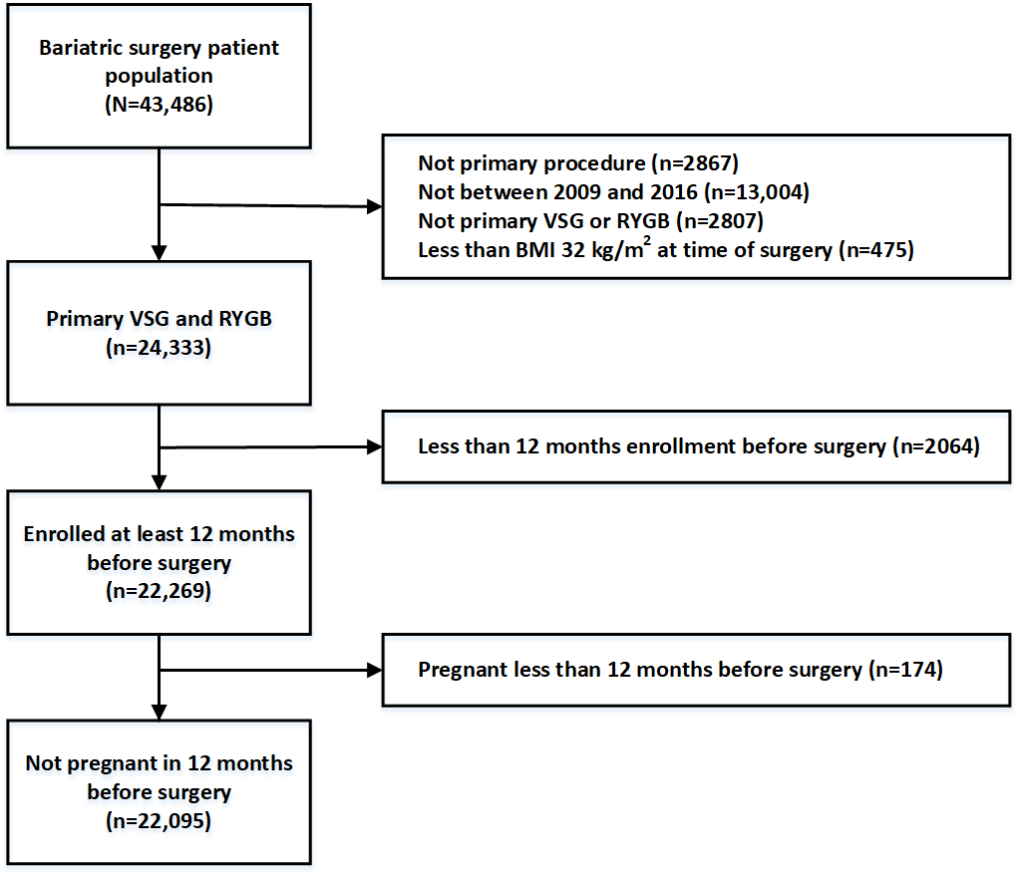
Inclusion and exclusion criteria for the ENGAGE CVD (Effectiveness of Gastric Bypass versus Gastric Sleeve for Cardiovascular Disease) cohort study. RYGB: Roux-en-Y gastric bypass; VSG: vertical sleeve gastrectomy.

Figure 1 shows the process of selecting the ENGAGE CVD cohort (n=22,095) and Table 1 presents descriptive statistics for the RYGB and VSG patients in the cohort. The cohort was assembled from 2009 to 2016 from a large integrated health care system serving the Southern California region of the United States. This health care system had 4.2 million members, 14 hospitals, 200 medical offices, 5700 physicians, and 23 bariatric surgeons at the time the cohort was assembled. Inclusion and exclusion criteria for the ENGAGE CVD cohort are shown in Figure 1.

This cohort of bariatric surgery patients was similar to other bariatric studies published in the United States, with the exception that there was a much higher proportion of ethnic/racial minorities (63.8%) than in other published work [17,18]. Eligibility for weight loss surgery in this health care system was based upon national recommendations [19]: Having a BMI ≥40 kg/m^2^ or having a BMI of 35-39 kg/m^2^ and at least one obesity-related comorbid condition such as sleep apnea, T2DM, and heart disease. Patients meeting these criteria could still be refused surgery if the surgeon determined that the patient had excessively high medical risk for surgery and in some cases, patients could have surgery if their BMI was as low as 32 kg/m^2^ with T2DM. Only 3.66% (808/22,095) of the ENGAGE CVD cohort had a BMI of 32-34.99 kg/m^2^ at the time of their operation.

### Measures

Bariatric surgery patients were followed up for the length of their membership in the health care system. Weight, height, and blood pressure were measured at every outpatient visit. In general, laboratory measures relevant to CVD such as glucose and glycated hemoglobin (HbA_1c_) were measured before surgery and at least annually following surgery. Lipids were only measured routinely every 5 years following national screening guidelines [20]. All data were abstracted from the electronic health record for the period of 2009 to 2018 and comprised the following broad categories of information.

#### Baseline

At the time of surgery, patient self-reported date of birth, gender, and race/ethnicity were obtained. Details of surgery type, surgeon, and surgery location were also assembled for the cohort.

#### Baseline and Follow-Up

Data were obtained for 24 months before the date of surgery and up to 10 years after surgery and included the following: (1) Dates and status of health care system enrollment and types of insurance coverage including pharmacy coverage; (2) vital signs such as height, weight, and blood pressure (in general, height was self-reported, and weight and blood pressure were measured by clinical staff at every outpatient visit. Previous research in health care settings has demonstrated that heights and weights from electronic medical records are valid and suitable for research [21]. Most blood pressure measurements were performed by certified medical assistants using automatic devices.); (3) self-reported smoking status from outpatient visits; (4) comprehensive prescription data for each drug dispensed at health care system pharmacies and all outpatient and inpatient laboratory results were also available (almost all patients [>96%] had benefits that incentivized the use of health care system pharmacies and laboratories); (5) all dates and types of health care utilization for inpatient, emergency department, and outpatient settings (including external claims data from contracted surgical providers); and (6) the diagnoses and procedures associated with this health care utilization.

### Outcomes

The primary outcome for the first aim of the ENGAGE CVD study was T2DM remission and relapse in each of the years of follow-up after bariatric surgery up to 5 years. Secondary outcomes for aim 1 were hypertension and dyslipidemia remission and relapse, and overall 10-year CVD risk as assessed with the new American College of Cardiology and the American Heart Association guidelines, referred to as the Pooled Cohort Equations Risk Calculator or ASCVD risk score [22], over this same time period. The primary outcome for aim 2 was a 30-day composite measure of major adverse events specific to bariatric surgery patients. The secondary outcomes for aim 2 were long-term annual rates of reoperations/revisions, readmissions, emergency department use, and all-cause mortality up to 5 years following bariatric surgery.

### Analyses

Summary statistics for the ENGAGE CVD cohort were generated using means and standard deviations for continuous variables and frequency and percent for categorical variables. Unadjusted differences between patients who were alive and still members of the health care system 5 years after surgery (n=13,774) and those patients who were not (n=2269), as well as between patients who had RYGB (n=8636) and VSG (n=13,459) were analyzed with independent sample t tests (continuous); and the Chi-square statistic and Kruskal-Wallis test (categorical).

The main analysis for the outcomes was a LIV approach [23]. This approach used a continuous instrumental variable to estimate the effect on every margin of the patient population and estimated population average effects to understand how different patients did with different treatments. This is referred to as heterogeneity of treatment effects (HTE) [24]. A clinically intuitive description of these methods applied to a clinical setting has been recently published [25]. These findings were compared with more traditional comparative effectiveness methods in retrospective observational studies, such as inverse-probability weighted propensity score regression [26], that only controlled for observed confounders in the decision between VSG and RYGB operations.

## Results

### Participants

Descriptive characteristics for patients in the ENGAGE CVD cohort are shown in Table 1. Overall, the cohort was 44.6 (SD 11.35) years old, with 80.19% (17,718/22,095) women, 18.94% (4185/22,095) non-Hispanic blacks, and 41.80% (9235/22,095) Hispanics. Patients had an average BMI of 44.30 (SD 6.88) kg/m^2^ at surgery with the majority having a BMI between 35-50 kg/m^2^ (17,386/22,095; 78.69%). In the 2 years before surgery, patients had been diagnosed with the following conditions: 36.56% (8078/22,095) gastroesophageal reflux disease (GERD), 15.48% (3421/22,095) sleep apnea, 36.72% (8114/22,095) T2DM, 53.80% (11,887/22,095) hypertension, 72.82% (16,090/22,095; dyslipidemia, and 11,967/22,095; 54.16% mental health condition (primarily depression). Only 3.44% (759/22,095) had a CVD event in their lifetime before surgery.

### Missing Data

Of the 22,095 patients in the ENGAGE CVD cohort, 16,043 (72.61%) had surgery between 2009 and 2013 and thus had enough follow-up time for the assessment of outcomes at 5 or more years following surgery. Of these 16,043 patients, 13,774 (85.86%) were still living (104 died before 5 years) and members of the health plan (2165 discontinued membership 5 years after surgery) at 5 years after surgery. Table 2 presents differences in baseline data for the ENGAGE CVD cohort of patients who were alive and still members of the health care system 5 years after surgery (n=13,774) compared with those patients who were not (n=2269). When compared with patients who did not contribute data to the 5-year outcome analysis for the ENGAGE CVD cohort (2269/16,043; 14.14%), patients who contributed data (13,774/16,043; 85.86%) were older (*P*=.002), more likely to be women (*P*=.02), more likely to be non-Hispanic white (*P*<.001), more likely to have a duodenal ulcer at the time of surgery (*P*<.001), less likely to have dyslipidemia (*P*<.001), more likely to have had an emergency department visit in the year before surgery (*P*=.006), less likely to have a mental illness before surgery (*P*<.001), and more likely to have had a CVD event at any time before surgery (*P*<.001).

**Table 1:** Characteristics of patients before surgery who are included in the ENGAGE CVD (Effectiveness of Gastric Bypass versus Gastric Sleeve for Cardiovascular Disease) cohort study (n=22,095) by bariatric operation (vertical sleeve gastrectomy [VSG] and Roux-en-Y gastric bypass [RYGB]. Characteristics at the time of surgery are compared between VSG and RYGB.

Characteristics	Overall (n=22,095)	VSG^a^ (n=13,459)	RYGB^b^ (n=8636)	*P* value
Age years), mean (SD)	44.6 (11.35)	44.1 (11.39)	45.4 (11.25)	<.001
Women, n (%)	17,718 (80.19)	10,850 (80.62)	6868 (79.53)	.06
Non-Hispanic black, n (%)	4185 (18.94)	2886 (21.44)	1299 (15.04)	<.001
Hispanic, n (%)	9235 (41.80)	5576 (41.43)	3659 (42.37)	<.001
Non-Hispanic white, n (%)	7997 (36.19)	4599 (34.17)	3398 (39.35)	<.001
Other, n (%)	678 (3.07)	398 (2.96)	280 (3.24)	<.001
Weight loss in the year before surgery (lbs), mean (SD)	−17.2 (14.74)	−17.8 (14.53)	−16.4 (15.02)	<.001
BMI at surgery (kg/m^2^), mean (SD)	44.3 (6.88)	43.8 (6.63)	45.1 (7.17)	<.001
BMI 32-34.99 kg/m^2^ at surgery, n (%)	808 (3.66)	547 (4.06)	261 (3.02)	<.001
BMI 35-39.99 kg/m^2^ at surgery, n (%)	5531 (25.03)	3633 (26.99)	1898 (21.98)	<.001
BMI 40-49.99 kg/m^2^ at surgery, n (%)	11,856 (53.66)	7185 (53.38)	4671 (54.09)	<.001
BMI >50 kg/m^2^ at surgery, n (%)	3900 (17.65)	2094 (15.56)	1806 (20.91)	<.001
Any lifetime cardiovascular disease event before surgery, n (%)	759 (3.44)	421 (3.13)	338 (3.91)	.002
Gastroesophageal reflux disease in 2 years before surgery, n (%)	8078 (36.56)	4472 (33.23)	3606 (41.76)	<.001
Esophagitis in 2 years before surgery, n (%)	388 (1.76)	217 (1.61)	171 (1.98)	.04
Gastric ulcer in 2 years before surgery, n (%)	153 (0.07)	96 (0.071)	57 (0.07)	.64
Duodenal ulcer in 2 years before surgery, n (%)	1411 (6.39)	816 (6.06)	595 (6.89)	.01
Peptic ulcer in 2 years before surgery, n (%)	346 (1.57)	197 (1.46)	149 (1.73)	.13
Gastritis duodenitis in 2 years before surgery, n (%)	2538 (11.49)	1518 (11.28)	1020 (11.81)	.23
Dyspepsia in 2 years before surgery, n (%)	2625 (11.88)	1614 (11.99)	1011 (11.71)	.52
Hiatal hernia in 2 years before surgery, n (%)	688 (3.11)	382 (2.84)	306 (3.54)	.003
Gastrointestinal bleed in 2 years before surgery, n (%)	9 (0.00)	5 (0.00)	4 (0.00)	.74
Aspirin use in 1 year before surgery, n (%)	3925 (17.76)	1875 (13.93)	2050 (23.74)	<.001
Aspirin use in 3 months before surgery, n (%)	2517 (11.39)	1255 (9.32)	1262 (14.61)	<.001
NSAID^c^ use in 1 year before surgery, n (%)	9630 (43.58)	5916 (43.96)	3714 (43.01)	.17
NSAID use in 3 months before surgery, n (%)	3260 (14.75)	1985 (14.75)	1275 (14.76)	.975
Cirrhosis in 2 years before surgery, n (%)	122 (0.01)	77 (0.01)	45 (0.01)	.62
Sleep apnea in 2 years before surgery, n (%)	3421 (15.48)	1983 (14.73)	1438 (16.65)	<.001
Type 2 diabetes mellitus in 2 years before surgery, n (%)	8114 (36.72)	3827 (28.43)	4287 (49.64)	<.001
Hypertension in 2 years before surgery, n (%)	11,887 (53.80)	6704 (49.81)	5183 (60.01)	<.001
Chronic kidney disease in 2 years before surgery, n (%)	2623 (11.87)	1402 (10.42)	1221 (14.14)	<.001
Dyslipidemia in 2 years before surgery, n (%)	16,090 (72.82)	9409 (69.90)	6681 (77.36)	<.001
Any mental health condition in 2 years before surgery, n (%)	11,967 (54.16)	7153 (53.15)	4814 (55.74)	<.001
Attendance rate in 1 year before surgery (range 0%-100%), mean (SD)	76.60 (12.39)	76.30 (12.31)	77.00 (12.49)	<.001
Any inpatient visit 1 year before surgery, n (%)	1317 (5.96)	722 (5.36)	595 (6.89)	<.001
Any emergency department visit in 1 year before surgery, n (%)	4655 (21.07)	2788 (20.71)	1867 (21.62)	.11

^a^VSG: vertical sleeve gastrectomy.

^b^RYGB: Roux-en-Y gastric bypass.

^c^NSAID: nonsteroidal anti-inflammatory drug.

**Table 2:** Characteristics of patients before surgery in the ENGAGE CVD (Effectiveness of Gastric Bypass versus Gastric Sleeve for Cardiovascular Disease) cohort study who accumulated 5 years of follow-up after surgery (n=16,043). Findings are compared for those patients who had missing (2269/16,043; 14.14%) and no missing (13,774/16,043; 85.86%) data at 5 years following bariatric surgery.

Variables	Accumulated 5 years of follow-up (N=16,043)	Missing 5-year data (N=2269)	Complete 5-year data (N=13,774)	*P* value
Roux-en-Y gastric bypass, n (%)	6891 (42.95)	1104 (48.66)	5787 (42.01)	<.001
Vertical sleeve gastrectomy, n (%)	9152 (57.05)	1165 (51.34)	7987 (57.99)	<.001
Age (years), mean (SD)	44.1 (11.92)	45.0 (9.54)	44.0 (12.26)	.002
Women, n (%)	12,860 (80.16)	1779 (78.40)	11,081 (80.45)	.02
Non-Hispanic black, n (%)	3067 (19.12)	375 (16.52)	2692 (19.54)	<.001
Hispanic, n (%)	6470 (40.33)	922 (40.64)	5548 (40.28)	<.001
Non-Hispanic white, n (%)	6011 (37.47)	929 (40.94)	5082 (36.90)	<.001
Other, n (%)	495 (3.09)	43 (1.90)	452 (3.28)	<.001
Weight loss in year before surgery (lbs), mean (SD)	−16.9 (15.01)	−17.3 (14.94)	−16.9 (15.02)	.04
BMI at surgery (kg/m^2^), mean (SD)	44.7 (6.95)	44.8 (7.17)	44.6 (6.91)	.59
BMI 32-34.99 kg/m^2^ at surgery, n (%)	508 (3.17)	69 (3.04)	439 (3.19)	.84
BMI 35-39.99 kg/m^2^ at surgery, n (%)	3701 (23.07)	516 (22.74)	3185 (23.12)	.84
BMI 40-49.99 kg/m^2^ at surgery, n (%)	8818 (54.96)	1243 (54.78)	7575 (55.00)	.84
BMI >50 kg/m^2^ at surgery, n (%)	3016 (18.80)	441 (19.44)	2575 (18.69)	.40
Any lifetime cardiovascular disease event before surgery, n (%)	759 (4.73)	0 (0.00)	759 (5.51)	<.001
Gastroesophageal reflux disease in 2 years before surgery, n (%)	5799 (36.15)	800 (35.26)	4999 (36.29)	.34
Esophagitis in 2 years before surgery, n (%)	281 (1.75)	33 (1.45)	248 (1.80)	.24
Gastric ulcer in 2 years before surgery, n (%)	108 (0.07)	18 (0.08)	90 (0.07)	.45
Duodenal ulcer in 2 years before surgery, n (%)	1089 (6.79)	108 (4.76)	981 (7.12)	<.001
Peptic ulcer in 2 years before surgery, n (%)	264 (1.65)	39 (1.72)	225 (1.63)	.77
Gastritis duodenitis in 2 years before surgery, n (%)	1767 (11.01)	240 (10.58)	1527 (11.09)	.47
Dyspepsia in 2 years before surgery, n (%)	1837 (11.45)	262 (11.55)	1575 (11.43)	.88
Hiatal hernia in 2 years before surgery, n (%)	476 (2.97)	71 (3.13)	405 (2.94)	.62
Gastrointestinal bleed in 2 years before surgery, n (%)	9 (0.00)	2 (0.00)	7 (0.00)	.49
Aspirin use in 1 year before surgery, n (%)	2999 (18.69)	412 (18.16)	2587 (18.78)	.48
Aspirin use in 3 months before surgery, n (%)	1900 (11.84)	245 (10.80)	1655 (12.02)	.10
NSAID^a^ use in 1 year before surgery, n (%)	6829 (42.57)	986 (43.46)	5843 (42.42)	.36
NSAID use in three months before surgery, n (%)	2342 (14.60)	346 (15.25)	1996 (14.49)	.34
Cirrhosis in 2 years before surgery, n (%)	84 (0.01)	12 (0.01)	72 (0.01)	.97
Sleep apnea in 2 years before surgery, n (%)	2330 (14.52)	346 (15.25)	1984 (14.40)	.29
Type 2 diabetes mellitus in 2 years before surgery, n (%)	5884 (36.68)	849 (37.42)	5035 (36.55)	.43
Hypertension in 2 years before surgery, n (%)	8768 (54.65)	1270 (55.97)	7498 (54.44)	.17
Chronic kidney disease in 2 years before surgery, n (%)	2170 (13.53)	319 (14.06)	1851 (13.44)	.42
Dyslipidemia in 2 years before surgery, n (%)	11,348 (70.73)	1844 (81.27)	9504 (69.00)	<.001
Any mental illness in 2 years before surgery, n (%)	8680 (54.10)	1304 (57.47)	7376 (5.355)	<.001
Attendance rate in 1 year before surgery (range 0%-100%), mean (SD)	76.70 (12.53)	76.50 (12.31)	76.70 (12.56)	.19
Any inpatient visit 1 year before surgery, n (%)	1081 (6.74)	159 (7.01)	922 (6.69)	.58
Any emergency department visit in 1 year before surgery, n (%)	3414 (21.28)	433 (19.08)	2981 (21.64)	.006

^a^NSAID: nonsteroidal anti-inflammatory drug.

### Understanding the Decisions Between Bariatric Operations

Table 1 presents pairwise comparisons between VSG and RYGB patients in the ENGAGE CVD cohort to highlight the importance of using state-of-the-art statistical and econometric comparative effectiveness methods to adjust for differences in patient populations between those who receive VSG and those who have RYGB [23-26]. VSG patients, when compared with RYGB patients in the ENGAGE CVD cohort, were younger (*P*<.001), were more likely to be of a racial and ethnic minority group (*P*<.001), lost more weight before surgery (*P*<.001), and had a lower BMI (*P*<.001); and were less likely to have a BMI >50 kg/m^2^ at the time of surgery (*P*<.001), had fewer lifetime CVD events (*P*=.002), and were less likely to be using aspirin before surgery (*P*<.001).

In addition, VSG patients when compared with RYGB patients in the ENGAGE CVD cohort had lower rates of GERD (*P*<.001), hiatal hernia (*P*=.003), sleep apnea (*P*<.001), T2DM (*P*<.001), hypertension (*P*<.001), chronic kidney disease (*P*<.001), dyslipidemia (*P*<.001), and mental illness (*P*<.001) at the time of surgery. Compared with RYGB patients, VSG patients had higher attendance rates for scheduled outpatient visits (*P*<.001) and lower rates of inpatient (*P*<.001) service use in the year before surgery.

As part of the process of understanding the decisions between bariatric operations, we conducted a series of meetings over 2 years with bariatric surgeons, patients, and providers about decisions they made between VSG and RYGB. We assembled a set of factors that our stakeholders felt were key determinants of why patients would undergo VSG or RYGB in Table 3. These factors were used to (1) construct propensity models with covariate adjustment and (2) test and select instrumental variables, which use natural variation to mimic random assignment to procedure, for comparative effectiveness analyses. Some of these variables, although important determinants of treatment assignment, were not included in our study because they were not available in the electronic health record. We included these variables in Table 3 because they illustrate the need to use statistical methods that can account for unmeasured confounders in the choice between bariatric operations. Most surgeons and providers indicated that patient preferences for one operation over another would be honored unless the operation they chose was a substantial safety risk for the patient.

**Table 3:** Factors considered as determinants in bariatric surgery decisions by a group of health care system stakeholders including patients, providers, and bariatric surgeons in the ENGAGE CVD (Effectiveness of Gastric Bypass versus Gastric Sleeve for Cardiovascular Disease) cohort study.

Factor	Preferred operation	Rationale	Available in electronic medical record
Year of surgery	Depends on year	Secular trends in surgery were apparent with RYGB^a^ preferred in years before 2011 and VSG^b^ preferred after 2011.	Yes
Preparation course instructor	Depends on instructor	Preparation course instructors have operation preferences and can communicate these to the patients and influence their choices.	Yes
Bariatric surgeon	Depends on surgeon	Surgeons have operation preferences as evidenced by frequency of type of operation over time.	Yes
Media consumption	Depends on source	Patients may be influenced to choose an operation based on electronic and other media consumption.	No
Patient race/ethnicity	VSG	More non-Hispanic black patients are having VSG compared with RYGB possibly because it is *less surgery,* and they will not lose *too much weight.*	Yes
History of cirrhosis and abdominal surgeries	VSG	Some bariatric surgeons believed that RYGB was inappropriate for patients with a history of cirrhosis and/or abdominal surgeries.	Yes
NSAID^c^ and aspirin use	VSG	Some bariatric surgeons believed that patients requiring anti-inflammatories (NSAIDs, aspirin, and steroids) were high risk for surgery regardless of operation type; however, the highest risk was for RYGB.	Yes
BMI >50 kg/m^2^	VSG	Some bariatric surgeons believed that much heavier patients had higher complication rates and that patients could be offered VSG to induce weight loss for a possible later, safer RYGB operation.	Yes
Medication-treated mental health	VSG	Some bariatric surgeons believed that patients requiring medication for mental health conditions may not do well after RYGB because of changes in absorption/metabolism after surgery.	Yes
Poor portion control	VSG	Some bariatric surgeons believed that if patients were severely obese mostly because of portion control, VSG would be the most conservative and successful option.	No
Complications	VSG	Most bariatric surgeons felt that VSG resulted in fewer complications than RYGB and should be the preferred operation to start, unless clearly contraindicated by GERD^d^ or gastrointestinal conditions.	Yes
Sweet eating/craving	RYGB	Some bariatric surgeons believed that the adverse consequence of *dumping syndrome* with RYGB following sweet-eating binges was a good deterrent for these patients helping them be more successful.	No
Type 2 diabetes mellitus, hiatal hernia, and GERD	RYGB	Some bariatric surgeons believed that RYGB was better for diabetes remission, and hiatal hernia and GERD would complicate VSG.	Yes

^a^RYGB: Roux-en-Y gastric bypass.

^b^VSG: vertical sleeve gastrectomy.

^c^NSIAD: nonsteroidal anti-inflammatory drug.

^d^GERD: gastroesophageal reflux disease.

## Discussion

### Principal Findings

The ENGAGE CVD cohort was one of the largest sample sizes of real-world bariatric operations, especially VSG, which is now the most common operation performed in the United States [8]. Randomized controlled trials (RCTs) do not have the sample size necessary to properly explore HTE, which can guide subgroups of patients in their decision whether to choose weight loss surgery as a treatment option and then which operation to have [12,15]. In addition, the ENGAGE CVD cohort had an excellent long-term follow-up. Nearly 85.86% (13,774/16,043) of patients were members of the health care system 5 years after surgery (see Table 2). Finally, unlike almost any study in the bariatric surgical literature, the ENGAGE CVD cohort was 64% non-white, providing a rare opportunity to study the effects of bariatric surgery for different racial and ethnic minorities, some of whom have the highest rates of severe obesity in the United States [1]. The ENGAGE CVD cohort has a bariatric surgery patient profile similar to that of the United States in the next 5 to 10 years, as nationwide bariatric practice shifts strongly toward VSG and the United States becomes more racially and ethnically diverse.

### Strengths and Weaknesses

The main weaknesses of the ENGAGE CVD cohort study were that all patients were insured, and although surgery was performed by 23 different surgeons across many settings, including surgeons outside of the health care system, the patients in the ENGAGE CVD cohort were cared for primarily within a single integrated health care system. This health care system may not be representative of the care, both preoperatively and postoperatively, that other patients might receive in different kinds of health care settings. In addition, the data were assembled retrospectively from electronic health records that were designed for clinical care and not research. Thus, data were not systematically collected by research personnel at regular intervals. Outcomes were not assessed in a standardized way by research personnel and had to be defined using methods that combined the clinical information available in the electronic health record with clinical stakeholder input about treatment guidelines and practices. There were no mechanisms for obtaining measures from patients who missed appointments and/or disenrolled from the health care system. Despite these limitations, we have shown that data from electronic medical records, such as heights and weights, are valid and suitable for research [21].

In addition, patients were not randomly chosen for surgery from an eligible pool of participants and they were not randomly assigned to operations. This threatens both internal validity (differences between operations could have been because of the assignment process) and external validity (those receiving bariatric operations were not representative of all the patients who were potentially eligible to have these operations). RCTs would be the best statistical design to evaluate the causal *efficacy* of bariatric surgery for cardiovascular risk reduction (highest internal validity) [27,28]. However, RCTs have poor external validity and cannot answer questions about what will work in an uncontrolled real-world setting or in a population more heterogeneous than the restrictive trial sample that is typically studied [29]. Retrospective observational comparative effectiveness cohort studies such as ENGAGE CVD are better designs for testing how well *existing efficacious* treatments work for a heterogeneous patient population in an uncontrolled real-world setting.

### Conclusions

The goal of the ENGAGE CVD study was to provide population-based, comprehensive, rigorous evidence for both clinical and policy decision making, informing the choice between RYGB and VSG for overall CVD risk reduction and risk factor remission, as well as safety in a diverse group of patients (racial and ethnic minority). Our findings will be used to provide recommendations to providers and patients about the decision between operations and help prioritize future health policy decisions and research investments in this area.

## Abbreviations

CVD: cardiovascular disease
ENGAGE CVD: Effectiveness of Gastric Bypass versus Gastric Sleeve for Cardiovascular Disease
GERD: gastroesophageal reflux disease
HTE: heterogeneity of treatment effects
LIV: local instrumental variable
RCT: randomized controlled trial
RYGB: Roux-en-Y gastric bypass
T2DM: type 2 diabetes mellitus
VSG: vertical sleeve gastrectomy
